# Delay in cART Initiation Results in Persistent Immune Dysregulation and Poor Recovery of T-Cell Phenotype Despite a Decade of Successful HIV Suppression

**DOI:** 10.1371/journal.pone.0094018

**Published:** 2014-04-07

**Authors:** Patricia Ndumbi, Julian Falutz, Nitika Pant Pai, Christos M. Tsoukas

**Affiliations:** 1 Immune Deficiency Treatment Centre, McGill University Health Centre, Montreal (Quebec), Canada; 2 Division of Clinical Epidemiology, McGill University Health Centre, Montreal (Quebec), Canada; Istituto Superiore di Sanità, Italy

## Abstract

**Background:**

Successful combination antiretroviral therapy (cART) increases levels of CD4+ T-cells, however this increase may not accurately reflect long-term immune recovery since T-cell dysregulation and loss of T-cell homeostasis often persist. We therefore assessed the impact of a decade of effective cART on immune regulation, T-cell homeostasis, and overall T-cell phenotype.

**Methods:**

We conducted a retrospective study of 288 HIV+ cART-naïve patients initiating therapy. We identified 86 individuals who received cART for at least a decade, of which 44 consistently maintained undetectable plasma HIV-RNA levels throughout therapy. At baseline, participants were classified into three groups according to pre-treatment CD4+ T-cell counts: Group I (CD4<200 cells/mm^3^); Group II (CD4: 200–350 cells/mm^3^); Group III (CD4>350 cells/mm^3^). Outcomes of interest were: (1) CD4+ T-cell count restoration (CD4>532 cells/mm^3^); (2) normalization of CD4:CD8 T-cell ratio (1.2–3.3); (3) maintenance of CD3+ T-cell homeostasis (CD3: 65%–85% of peripheral lymphocytes); (4) normalization of the complete T-cell phenotype (TCP).

**Results:**

Despite a decade of sustained successful cART, complete T-cell phenotype normalization only occurred in 16% of patients, most of whom had initiated therapy at high CD4+ T-cell counts (>350 cells/mm^3^). The TCP parameter that was the least restored among patients was the CD4:CD8 T-cell ratio.

**Conclusions:**

Failure to normalize the complete T-cell phenotype was most apparent in patients who initiated cART with a CD4+ T-cell count <200 cells/mm^3^. The impact of this impaired T-cell phenotype on life-long immune function and potential comorbidities remains to be elucidated.

## Introduction

The immune system of healthy individuals is characterized by the maintenance of homeostasis via a balanced T-cell phenotype (TCP) [1]. Although the hallmark of HIV infection is progressive CD4+ T-cell depletion, other impairments in immune phenotype such as loss of T-cell homeostasis and severe T-cell subset dysregulation also occur.

T-cell subset dysregulation was the earliest noted surrogate marker of AIDS in the early 1980s [2], [3]. It is characterized by a low CD4:CD8 T-cell ratio (<1.2) resulting from the depletion of CD4+ T-cells and the concomitant expansion of the CD8+ population of T-cells in the peripheral blood. Studies have shown that in HIV seropositive individuals receiving long-term combination antiretroviral therapy (cART), CD4:CD8 T-cell ratio dysregulation is correlated with higher risk of developing coronary artery disease [4]. In non-HIV settings, low CD4:CD8 T-cell ratios are also associated with poor clinical outcomes in patients with common variable immune deficiency (CVID) and in healthy individuals over the age of 60 [5], [6].

T-cell homeostasis was first described in 1993 by Adleman and Wofsy as the normal physiologic state by which the immune system maintains a constant number of circulating CD3+ T-cells, irrespective of changes within the CD4+ and CD8+ T-cell compartments [7]. Loss of T-cell homeostasis often occurs in HIV-infected individuals, and is manifested by a failure to maintain normal levels of circulating CD3+ T-cells [8]. Data from 372 seroconverters enrolled in the Multicenter AIDS Cohort Study (MACS) showed that in the absence of adequate treatment, the loss of T-cell homeostasis in HIV infection could predict impending AIDS and death [9]. We recently showed for the first time that impairment in T-cell homeostasis is also associated with morbidity and mortality among HIV-positive patients who receive effective combination antiretroviral therapy (cART) [10]. In non-HIV clinical contexts, altered T-cell homeostasis has also been linked with deleterious clinical disorders such as rheumatoid arthritis, Crohn’s disease and systemic lupus erythematosus [11], [12]. The triad of low CD4+ T-cell count, dysregulated CD4:CD8 T-cell ratio, and loss of T-cell homeostasis characterizes the abnormal T-cell phenotype induced by HIV infection [3], [13].

The goals of effective cART are to suppress HIV viral replication and restore immune competence. Successful cART usually results in increased CD4+ T-cell counts, yet the increase may not reflect a complete immune recovery since ratio dysregulation and altered T-cell homeostasis often persist [13]–[16]. Failure to recover a balanced T-cell phenotype may put HIV-infected patients at risk for non-viral morbidities despite cART-mediated viral control [4], [10], [17], [18]. However, there is a scarcity of information on the effect of long-term suppressive cART on the normalization of the TCP. It is currently unknown whether this parameter can be completely restored in successfully treated individuals.

A major impediment to the evaluation of true immune recovery among HIV-positive patients is the limited number of surrogate markers available for clinical use. Measuring the total number of circulating CD4+ T-cells often necessitates the simultaneous determination of CD8+ and CD3+ T-cell numbers. Therefore, monitoring CD3+ T-cell levels and CD4:CD8 T-cell ratio, in addition to CD4+ T-cell counts, may provide further insight into immune restoration. Due to the availability of more effective and better tolerated antiretrovirals (ARVs), HIV-positive patients are now able to achieve sustained viral suppression over longer periods of time, thus allowing for the evaluation of very long-term immune recovery. In this pilot study, we assessed the extent of T-cell phenotype recovery among a group of treated HIV-positive males who achieved and maintained viral suppression for at least a decade.

## Methods

### Ethics Statement

Institutional approval for this study was obtained from the Research Ethics Board (REB) of the Montreal General Hospital. The REB approves the anonymous use of data retrospectively abstracted from clinical care databases without requiring patient consent. Patients sign a general waiver upon opening a medical chart.

### Cohort Description

We conducted a retrospective cohort analysis of a treatment-naïve group of 288 HIV-positive individuals followed at the Montreal General Hospital. Patients initiated cART as of 01-Jan-1995. Combination ART was defined as a combination of at least three anti-HIV drugs: either two nucleoside reverse transcriptase inhibitors (NRTIs) in combination with a non-NRTI (NNRTI) or a protease inhibitor (PI), two PIs in combination with at least one NRTI; or a combination of one PI, one NNRTI, and at least one NRTI. All patients had regular follow-up at three month intervals. At each follow-up, clinical, virologic, and immune evaluations were performed including longitudinal documentation of CD4:CD8 T-cell ratios as well as CD4+, CD8+, and CD3+ T-cell counts and percentages. Each study participant had a minimum follow-up of ten years from the time of cART initiation.

### Participant Selection

*Inclusion criteria:*

- Adults (>18 years of age) and of male gender.
- Date of cART initiation.
- Documented therapy with cART for a minimum of ten years.
- Complete adherence with cART and trimestrial clinic visits.
- Confirmation of viral suppression at each visit (<50 copies/mL).
- Clinical and immune phenotype data available during the entire follow-up period.

*Exclusion criteria:*

- Participants with incomplete baseline and follow-up data.
- Participants with clinical loss to follow-up exceeding twelve months.
- Participants with more than two consecutive viral load blips throughout their follow-up.

### Outcomes

The primary outcome of interest was the restoration of a balanced T-cell phenotype defined as meeting all six of the following criteria: CD3+ T-cell percent: 65%–85%; CD4:CD8 T-cell ratio 1.2–3.3; CD4+ T-cell count: 532–1170 cells/mm^3^; CD4+ T-cell percent: 39%–55%; CD8+ T-cell count 236–651 cells/mm^3^; CD8+ T-cell percent 18%–31%. These ranges represent the mean ±2 standard deviations and were derived from 124 healthy age-matched controls recruited at the Montreal General Hospital. These controls were all HIV-negative, had normal physical examinations, and were also screened for the presence of primary immune deficiencies and autoimmune diseases.

The primary outcome was chosen based on the known alterations in T-cell phenotype induced by HIV-1 infection that include low CD4+ T-cells, loss of CD3+ T-cell homeostasis, and a low CD4:CD8 T-cell ratio. Using our established normal ranges, the cutoffs for these parameters were as follows: low CD4+ T-cells (<532 cells/mm^3^); loss of T-cell homeostasis (low <65% or high >85%) CD3+ T-cell percentages; low CD4:CD8 T-cell ratio (<1.2). Since the sum of the CD4+ and CD8+ T-cell percentages proportionally define the T-cell compartment of circulating lymphocytes and are also used to generate the CD4:CD8 T-cell ratio, we also assessed the percentages of these subsets. The subset percentages are also not affected by fluctuations in lymphocyte numbers and their use ensured a thorough evaluation of the TCP.

### Statistical Analysis

Patients were assigned based on their baseline pre-treatment CD4+ T-cell counts as follows: Group I–CD4+ T-cell count <200 cells/mm^3^; Group II–CD4+ T-cell count = 200–350 cells/mm^3^; Group III–CD4+ T-cell count >350 cells/mm^3^. Patient characteristics were compared according to the baseline CD4+ T-cell group, and differences were assessed using the Kruskal-Wallis test for continuous variables and Fisher’s Exact test for categorical variables. Linear mixed effects models to account for correlation between repeated measurements within each individual, assuming an autoregressive of order 1 covariance structure, were used to estimate the annual rate of change in CD4+ T-cell count, CD4:CD8 ratio, and CD3+ T-cell percentage. An analysis of the residuals on each model suggested the need for a transformation of the outcome data in order to meet the assumptions. Thus in the final model, rates of change in CD4+ T-cell counts were estimated using square-root transformed absolute CD4+ T-cell values, which provides variance stabilization for repeated measures and has been previously used for CD4+ T-cell trajectory analysis [19], [20]. For the same reasons, the rates of change in CD4:CD8 T-cell ratios were estimated using natural logarithm transformed data. No transformation was required for the CD3+ T-cell percentages. The final model was adjusted for baseline age, CMV, and baseline HIV viral load (log10). All statistical hypothesis tests were two-sided and performed at the 0.05 level of significance. All analyses were done using the SAS software, version 9.2 (SAS Institute Inc. Cary, NC).

## Results

### Characteristics of the Study Population

From the original cART-naïve group of 288 HIV+ patients, we identified 86 males (29.9%) who remained consistently on cART for at least 10 years without any treatment interruptions. Among these 86 males, 44 (51.2%) met the inclusion criteria. These individuals achieved and maintained plasma HIV-RNA under the level of detection (<50 copies/ml) throughout their entire treatment time. The number of participants per group was 19, 14, and 11 for Group I, Group II, and Group III, respectively. The median time from HIV diagnosis to treatment initiation was six years (IQR: 2–10) and there was no significant difference between the groups. All patients were initiated on a 2 NRTI (3TC, AZT, or d4T) plus 1 PI (indinivir, saquinavir, or ritonavir) regimen, except for one patient in Group I who was on a 2 NRTI plus 1 NNRTI (nevirapine) regimen.

At baseline, the mean age of the patients was 42 years (IQR: 38.5–49.5), all patients were male, 57% were men who have sex with men (MSM), 75% were CMV seropositive, and 20% presented with an AIDS defining illness (ADI). The mean baseline HIV-RNA was 4.5 log_10_ copies/ml (IQR: 3.8–4.9), and the median duration of HIV treatment with cART was 14 years (IQR: 13–14). Baseline characteristics are summarized in Table 1.

**Table 1:** Demographics and clinical characteristics for 44 HIV-positive patients on combination antiretroviral therapy.

Characteristics	All Patients (n = 44)	Group I (n = 19)	Group II (n = 14)	Group III (n = 11)	P-value
**Age**	42 (39–50)	44 (35–58)	44(38–54)	42(35–44)	0.46
**HIV risk factor**					
Hemophiliac/blood	13	7	4	2	
MSM	25	10	8	7	
Heterosexual	6	2	2	2	
**Caucasian**	42 (95%)	17 (89%)	14 (100%)	11 (100%)	0.14
**Baseline VL (log_10_ copies/mL)**	4.5 (3.8–4.9)	4.8 (4.6–5.1)	3.8 (3.2–4.9)	4.1 (3.3–4.4)	0.14
**Nadir CD4 counts (cells/mL)**	130 (30–268)	30 (20–70)	178 (77–293)	308 (231–350)	0.0001
**ADI at baseline**	9 (20%)	6 (32%)	2 (14%)	1 (9%)	0.12
**HCV co-infection**	13 (30%)	6 (32%)	4 (29%)	3 (27%)	0.79
**CMV co-infection**	33 (75%)	16 (84%)	11 (79%)	6 (55%)	0.08
**Cardiovascular Events**	11 (25%)	6 (32%)	5 (36%)	0 (0%)	0.08
**Caucasian**	42 (95%)	17 (89%)	14 (100%)	11 (100%)	0.14

Values are number and percentage, or median and interquartile range. Group I: CD4+ T-cell count <200 cells/mm^3^; Group II: CD4+ T-cell count = 200–350 cells/mm^3^; Group III: CD4+ T-cell count >350 cells/mm^3^. MSM, men who have sex with men; VL, viral load; ARV, antiretroviral; ADI, AIDS defining illness; HCV, hepatitis C virus; CMV, cytomegalovirus.

### Normalization Of The T-Cell Phenotype And Clinical Outcomes

#### Cd4+ T-Cell Count

At baseline, only 2 of the 44 patients had a normal CD4+ T-cell count. These patients belonged to Group III. After a decade of suppressive cART, only 50% of Group I patients normalized their CD4+ T-cell count, while 86% of Group II, and 100% of Group III patients recovered this parameter (Table 2). Linear regression analysis showed that the rate of change in square root CD4+ T-cell counts was significantly higher in Group I (2.19/year, 95% CI = 1.47–2.92) compared to Group II (1.39/year, 95% CI = 0.64–2.16) and Group III (1.08/year, 95% CI = 0.41 to 1.75) within the first 5 years of treatment. In the subsequent 5 years, the annual change in square root CD4+ T-cell counts had decreased across the three groups (Group I: 0.64/year, 95% CI = 0.23–1.05; Group II: 0.57/year, 95% CI = 0.13–1.01, and Group III: 0.77/year, 95% CI = 0.38–1.15). However, there were no significant differences across the groups (Table 3).

**Table 2:** Proportion of patients with normal T-cell parameters at baseline and year 10.

Baseline					
**T-cell parameters**	**All (n = 44)**	**Group I (n = 19)**	**Group II (n = 14)**	**Group III (n = 11)**	**P-value**
CD3+ T-cell percent	33 (75)	13 (68)	10 (71)	10 (91)	0.37
CD4+ T-cell count	2 (5)	0	0	2 (18)	0.06
CD4:CD8 T-cell ratio	2 (5)	0	0	2 (18)	0.06
TCP	1 (2)	0	0	1 (10)	0.25
**Year 10**					
**T-cell parameters**	**All (n = 44)**	**Group I (n = 19)**	**Group II (n = 14)**	**Group III (n = 11)**	**P-value**
CD3+ T-cell percent	35 (80)	14 (73)	11 (78)	10 (91)	0.57
CD4+ T-cell count	33 (75)	10 (52)	12 (86)	11 (100)	0.007
CD4:CD8 T-cell ratio	16 (36)	2 (11)	6 (43)	8 (73)	0.002
TCP	7 (16)	1 (5)	1 (7)	5 (45)	0.02

Data are presented as n (%).

**Table 3:** Estimated differences in slope of T-cell parameters between groups based on multivariate linear mixed effect models.

Years 0–5	Parameters	Groups	Slope Difference	P-value
	CD4	Group 1–3	1.12	0.003
		Group 2–3	0.32	0.41
		Group 2–1	−0.8	0.02
	CD4:CD8	Group 1–3	0.16	0.002
		Group 2–3	0.02	0.74
		Group 2–1	−0.14	0.004
	CD3	Group 1–3	0.92	0.37
		Group 2–3	−0.48	0.66
		Group 2–1	−1.4	0.14
**Years 6–10**	**Parameters**	**Groups**	**Slope Difference**	**P-value**
	CD4	Group 1–3	−0.13	0.75
		Group 2–3	−0.2	0.65
		Group 2–1	−0.07	0.85
	CD4:CD8	Group 1–3	0.05	0.19
		Group 2–3	0.04	0.32
		Group 2–1	−0.01	0.79
	CD3	Group 1–3	−0.22	0.77
		Group 2–3	−0.09	0.91
		Group 2–1	0.13	0.85

Data represented are square root CD4 counts, geometric mean CD4:CD8 ratio, and mean CD3+ T-cell percent.

#### Cd4:cd8 T-Cell Ratio

**Figure 1:**
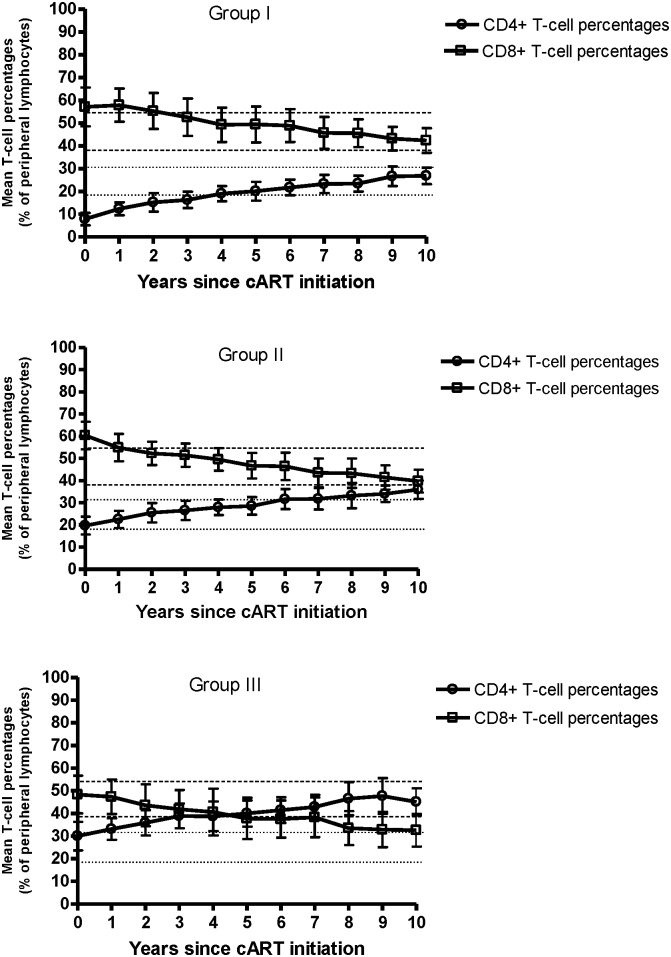
Longitudinal evaluation of the CD4:CD8 T-cell ratio from the time of cART initiation. Mean values of circulating CD4+ and CD8+ T-cell percentages as a function of years since cART initiation in Groups I, II, and III. Vertical bars indicate the 95% confidence intervals. The horizontal dashed lines represent upper and lower limits of the normal reference range for CD4+ T-cell percentages, and the horizontal dotted lines represent upper and lower limits of the normal reference range for CD8+ T-cell percentages.

At treatment initiation, the CD4:CD8 T-cell ratio was dysregulated in all patients except for two individuals in Group III. By year 10, the proportion of patients with normalized CD4:CD8 T-cell ratio was highest in Group III (73%) and lowest in Group I (11%). Less than half of Group II (43%) normalized this parameter (Table 2). Linear regression analysis showed that similarly to CD4+ T-cell counts, the annual increase in CD4:CD8 T-cell ratio within the first five years of treatment was highest in Group I (0.27/year, 95% CI = 0.17–0.37) compared to Group II (0.13/year, 95% CI = 0.03–0.23) and Group III (0.11/year, 95% CI = 0.02–0.21). However, no difference in the annual rate of change was observed during the last five years across the groups: Group I: 0.09/year, 95% CI = 0.04–0.14; Group II: 0.08/year, 95% CI = 0.02–0.14; Group III: 0.04/year, 95% CI  =  −0.01–0.09 (Table 3). A descriptive representation of the CD4+ and CD8+ T-cell percentage trajectories showed that the CD4:CD8 T-cell ratio imbalance was only successfully reversed in Group III (Figure 1).

#### Cd3+ T-Cell Homeostasis

Despite differences in T-cell subset balance, the proportion of patients with normal T-cell homeostasis at baseline and at follow-up was similar across the groups (Table 2). Linear regression analysis showed that there was no significant difference in the annual rate of change in CD3+ T-cell percentage across the three groups during the first half (Group I: 0.90/year, 95% CI  =  −1.08 to 2.88; Group II: −0.5/year, 95% CI  =  −2.62 to 1.62; Group III: −0.02/year, 95% CI  =  −1.86 to 1.82), and the last half of the follow-up (Group I: −0.23/year, 95% CI  =  −1.32 to 0.86; Group II: −0.1/year, 95% CI  =  −1.26 to 1.06; Group III: −0.02/year, 95% CI  =  −1.03 to 0.99) (Table 3).

#### Complete T-Cell Phenotype

Only 16% of all patients had a completely normalized TCP by the end of the follow-up period. Comparing individual groups, we observed that 45% of individuals in Group III achieved TCP normalization while only 7% of Group II and 5% of Group I normalized (Table 2). Overall, among the 44 patients, we found that the TCP parameter with the lowest normalization rate was the CD4:CD8 T-cell ratio with only 36% of all patients normalizing this parameter after a decade of suppressive therapy, 50% of those restoring this parameter belonged to Group III.

#### Clinical Outcomes

Throughout the course of the follow-up, 11 patients (25%) developed a cardiovascular event (CVE), as defined by the occurrence of an acute coronary syndrome (myocardial infarction, diagnosed unstable angina, or stroke). All of these individuals were co-infected with CMV. Overall, 32% of Group I and 36% of Group II developed a CVE. However, no CVE was observed among Group III patients (Table 1). After ten years of follow-up, only three deaths occurred within the sample: 2 (11%) in Group I and 1 (7%) in Group II. There were no reported deaths among Group III patients.

## Discussion

In the very first description of AIDS in 1981, the authors described a new acquired severe immune deficiency that was characterized by a loss of CD4+ T-cells (Leu 3+), a concomitant increase in CD8+ T-cells (Leu 2+), resulting in a low CD4:CD8 T-cell (Leu3+/Leu2+) ratio, and a severe loss of CD3+ T-cell (Leu 1+) homeostasis [3]. In this study, we describe results obtained from a cohort of 44 well-defined HIV-positive patients with long-term complete viral suppression following cART initiation. We detected a distinct subset of individuals, primarily among patients who initiated antiretroviral therapy at very low CD4+ T-cell counts, who failed to normalize key T-cell phenotype parameters.

The CD4+ T-cell count is currently the preferred marker for monitoring HIV progression, as low levels of CD4+ T-cells are associated with high risk of co-morbidities and mortality in HIV patients [21],[22]. Our findings show that despite the fact that three quarters of the population achieved a normal CD4+ T-cell count by the end of the follow-up, only about a third of the patients had a normal CD4:CD8 T-cell ratio at year ten. This suggests that recovery of CD4+ T-cell numbers does not always reflect the normalization of other T-cell phenotype parameters. Interestingly, our longitudinal analysis showed that Group I exhibited the greatest fold-increase in CD4+ T-cell counts within the first five years of treatment. However, this recovery rate slowed down in the second half of the follow-up, resulting in subnormal levels of CD4+ T-cells at the end of the follow-up despite continuous viral suppression. Our findings are in line with previous studies showing that HIV-positive patients initiating therapy at advanced stages of the disease experience important increases in CD4+ T-cell counts during the early phase of therapy, but exhibit substantially reduced annual increases during the rest of the follow-up [20],[23]–[25]. Several studies have suggested that the initial spike observed within circulating CD4+ T-cells during early treatment results from a redistribution of memory T-cells from lymphoid tissues to the periphery, rather than from de novo synthesis [26]. T-cell regenerative processes might thus be severely or even irreversibly impaired in patients who initiate therapy at advanced stages of the infection.

Among the 44 patients, we found that the parameter that was the least conserved was the CD4:CD8 T-cell ratio. Failure to restore a normal CD4:CD8 T-cell ratio has been linked with an increased risk of non-AIDS related events, such as cardiovascular disease [4]. Furthermore, a low CD4:CD8 T-cell ratio is an important component of the immune risk phenotype (IRP), which is associated with increased morbidity and mortality in seronegative individuals over the age of 60 [27],[28]. The inability to restore the ratio could similarly put HIV-positive patients at risk for non-AIDS mortality and comorbidities. Interestingly, a recent study by the Canadian Observational Cohort Collaboration (CANOC) has shown that T-cell ratio normalization might be associated with decreased risk of AIDS defining illnesses and mortality among treated patients [29]. Despite these findings, there is still a paucity of data available on long-term CD4:CD8 T-cell ratio recovery in patients receiving efficacious HIV therapy. Our study shows that among successfully treated HIV patients, T-cell ratio normalization is most frequent in those who initiate therapy at CD4+ T-cell counts above 350 cells/mm^3^. Despite over a decade of HIV suppression, more than half of Group II and most of Group I maintained persistently low ratios. A descriptive representation of CD4+ and CD8+ T-cell subset trajectories show that the ratio normalization observed in Group III is the result of both an increase in CD4+ T-cell levels and a concomitant decrease in CD8+ T-cell levels (Figure 1). This is a clear reversal of the post HIV seroconversion T-cell subset trend previously reported in cART-naive HIV-patients [9]. CD8+ T-cell percentages remained abnormally high in Groups I and II throughout the follow-up. Since all patients maintained HIV suppression, it is unlikely that HIV replication would account for the disproportionate CD8+ T-cell expansion observed in these two groups. It is possible that co-morbid chronic viral infections, such as cytomegalovirus (CMV) infection, contribute to CD8+ T-cell lymphocytosis [30]. CMV, a ubiquitous herpes virus, establishes a lifelong latent infection in humans [31],[32]. Naeger et al found that CMV-specific CD8+ T-cell responses are high in successfully treated HIV-positive patients [33]. Interestingly, CMV prevalence was highest among Group I and Group II patients. The persistent CD8+ lymphocytosis and low CD4:CD8 T-cell ratio observed in these groups might thus be partially explained by a subclinical CMV infection. Future studies are required to investigate the nature of the CMV immune response in such individuals.

We also noted that a quarter of our patient population developed cardiovascular events (CVE) throughout the follow-up period, however none of these patients belonged to Group III. The occurrence of CVE among Group I and II patients is particularly interesting considering that these two groups had the highest prevalence of CMV seropositivity. In fact, all of the eleven patients who experienced a cardiovascular event were co-infected with CMV. Although the clinical outcomes associated with acute CMV infection have been the subject of considerable investigation, the long-term impact of chronic asymptomatic CMV infection still needs to be elucidated. Considerable evidence seems to indicate an association between CMV seropositivity and an increased risk of cardiovascular morbidity via the induction of a pro-inflammatory response [34],[35].

Numerous reports of untreated individuals revealed that failure of T-cell homeostasis is an important landmark in HIV disease progression [8],[9],[36],[37]. We recently showed for the first time that even in those receiving potent cART, lost T-cell homeostasis was associated with morbidity and mortality [10]. However, to our knowledge, studies investigating long-term changes in circulating CD3+ T-cells in those with well-controlled HIV replication remain scarce. Our findings indicate that despite a high rate of CD4:CD8 T-cell dysregulation, most patients maintained normal CD3+ T-cell levels at the end of the follow-up. Therefore, CD3+ T-cell percentages remained relatively constant within each group despite drastic shifts in the CD4+ and CD8+ T-cell subsets as shown in Figure 1. These results are in accordance with previous findings in AIDS free HIV-infected individuals, where little variations were observed in CD3+ T-cell levels regardless of substantial changes in the CD4+ and CD8+ compartments [9]. This is in agreement with the concept of blind T-cell homeostasis, and indicates the existence of a physiologic mechanism that strives to maintain constant T-cell numbers in the face of intra-subset fluctuations. This phenomenon has also been observed in HIV-negative individuals, where CD3+ T-cell percentages were shown to be stable despite low (<1) or high (>1) CD4:CD8 T-cell ratios [27]. Although some studies have measured T-cell homeostasis in terms of CD3+ T-cell counts, in this study it was defined in terms of CD3+ T-cell percentages as we consider this parameter to be a more reliable marker of the proportional stability of the circulating T-cell pool. Indeed, T-cell percentages vary less than absolute counts since they are not affected by lymphocyte fluctuations [38]–[40].

Finally, the complete TCP was only restored in a small number of patients (16%), most of which had initiated treatment at high baseline CD4+ T-cell counts (>350 cells/mm^3^). Whether the other patients will eventually normalize TCP with longer treatment time remains unknown. It is unlikely that the type of treatment regimen used had a significant impact on the outcomes, since all but one patient were on a PI-based regimen. The clinical importance of a multiparametric assessment of T-cell recovery was also underlined in a study by Torti et al. [41]. The T-cell markers evaluated in that study included CD4+ T-cell counts, CD4+ T-cell percentages, and CD4:CD8 T-cell ratios. We feel that the inclusion of the CD3+ T-cell percentage in our analysis provides a more comprehensive measure of multiparametric T-cell recovery as it takes into consideration both CD4+ and CD8+ T-cell levels. Furthermore, our study had a follow-up period of 10 years (vs. a median of 4 years in Torti et al.), which to our knowledge, is the longest evaluation of a multiparametric T-cell phenotype recovery in HIV patients with consistently sustained viral suppression to levels below 50 copies/mL.

The current pilot study has a few limitations. First, we restricted our analysis to males because of the limited number of females being followed in our clinic, thus optimizing the homogeneity of the study population. Our results may therefore not apply to HIV-positive women. Secondly, the sample size of our study population was limited by the small size of our cohort and the rigorousness of our selection criteria. Studies using larger cohorts are therefore needed to validate these findings. It was previously demonstrated that early cART initiation can improve immunological outcomes presumably through a reduction of T-cell activation while on-therapy [42]. However, we could not assess the level of immune activation in each group, since inflammation markers are not usually collected from our patients during routine clinical visits. Finally, due to the small proportion of patients who achieved TCP normalization, the impact of the TCP on clinical outcomes could not be evaluated.

Treatment for HIV is life-long. Therefore, long-term and comprehensive evaluation of immune recovery is important. Historically, T-cell phenotypic recovery in HIV-infected patients was assessed by monitoring CD4+ T-cell counts. The originality of our work stems from the fact that this is the first study looking at an aggregate of specific T-cell phenotypic markers (CD4+ T-cell counts, CD4:CD8 T-cell ratio, and CD3+ T-cell percentages) in order to investigate immune recovery from an allostatic perspective. The rationale for the choice of these parameters is based on their individual association with poor clinical outcomes in both HIV and non-HIV settings. Although the sample size was small, an important strength of our study lies in the long duration of the follow-up period and requirement that all subjects maintained viral load levels below 50 copies of HIV-1 RNA at all time points. These study criteria allowed us to investigate long-term patterns of immune recovery in the context of highly effective viral suppression.

In this pilot study, the analysis was specifically focused on patients with prolonged and optimal viral suppression, as this represents the ideal clinical context for long-term immune recovery. Our findings show that normalization of the T-cell phenotype is best achieved when cART is initiated at baseline CD4+ T-cell counts >350 cells/mm^3^. Furthermore, despite increases in CD4+ T-cell counts, very few patients who initiate therapy at advanced stages of HIV infection recover a normal T-cell phenotype, even after a decade of therapy. Overall, our findings indicate that HIV infection causes profound TCP alterations that are not completely reversed with treatment. Larger cohort studies will be important to determine the long-term clinical impact of this persistent dysregulation, in order to assess whether an altered TCP is a risk profile associated with higher morbidity and mortality among HIV-infected patients.
